# Aging Effects and Test–Retest Reliability of Inhibitory Control for Saccadic Eye Movements

**DOI:** 10.1523/ENEURO.0459-19.2020

**Published:** 2020-09-28

**Authors:** Martyna Beata Płomecka, Zofia Barańczuk-Turska, Christian Pfeiffer, Nicolas Langer

**Affiliations:** 1 Methods of Plasticity Research, Department of Psychology, University of Zurich, CH-8050 Zurich, Switzerland; 2 Institute of Mathematics, University of Zurich, CH-8057 Zurich, Switzerland; 3 University Research Priority Program (URPP) Dynamic of Healthy Aging, CH-8050 Zurich, Switzerland; 4 Neuroscience Center Zurich (ZNZ), CH-8057 Zurich, Switzerland; 5 Center for Reproducible Science University of Zurich Hirschengraben 84, 8001 Zurich, Switzerland

**Keywords:** aging, antisaccade task, eye-tracking, inhibitory control, test–retest

## Abstract

Neuropsychological studies indicate that healthy aging is associated with a decline of inhibitory control of attentional and behavioral systems. A widely accepted measure of inhibitory control is the antisaccade task that requires both the inhibition of a reflexive saccadic response toward a visual target and the initiation of a voluntary eye movement in the opposite direction. To better understand the nature of age-related differences in inhibitory control, we evaluated antisaccade task performance in 78 younger (20–35 years) and 78 older (60–80 years) participants. In order to provide reliable estimates of inhibitory control for individual subjects, we investigated test–retest reliability of the reaction time, error rate, saccadic gain, and peak saccadic velocity and further estimated latent, not directly observable processed contributing to changes in the antisaccade task execution. The intraclass correlation coefficients (ICCs) for an older group of participants emerged as good to excellent for most of our antisaccade task measures. Furthermore, using Bayesian multivariate models, we inspected age-related differences in the performances of healthy younger and older participants. The older group demonstrated higher error rates, longer reaction times, significantly more inhibition failures, and late prosaccades as compared with young adults. The consequently lower ability of older adults to voluntarily inhibit saccadic responses has been interpreted as an indicator of age-related inhibitory control decline. Additionally, we performed a Bayesian model comparison of used computational models and concluded that the Stochastic Early Reaction, Inhibition and Late Action (SERIA) model explains our data better than PRO-Stop-Antisaccade (PROSA) that does not incorporate a late decision process.

## Significance Statement

The antisaccade task, widely used in the study of inhibitory control, offers a window onto the operation of executive functioning. This study established that the measures proposed by the internationally standardized antisaccades protocol are reliable over time and therefore constitute meaningful and suitable estimates for future longitudinal studies and identifying promising biomarkers for cognitive decline. Furthermore, older subjects exhibited longer saccadic reaction times and significantly higher average error rates. We further decomposed the task with computational models. We expanded previous findings by showing that aging differences in reaction time and error rate can be explained by fast or slow inhibition and the probability of generating late voluntary prosaccades.

## Introduction

Over the last decades, life expectancy has steadily increased and is predicted to further increase in the coming years (Kanasi et al., 2016). Although age-related changes in cognitive functions, such as executive control, attention, and memory, have been repeatedly demonstrated (for review, see Verhaeghen and Cerella, 2002; Rey-Mermet and Gade, 2018), the underlying processes remain largely unknown.

An executive function that is particularly affected by aging is inhibitory control, the ability to suppress highly practiced responses in favor of more appropriate reactions given the current context or goals (Connelly and Hasher, 1993; Houx and Jolles, 1993; Spieler et al., 1996; Crawford et al., 2005; Butler and Zacks, 2006; Rey-Mermet and Meier, 2017). Recently, the voluntary control of eye movement has been proposed as a simple to use, non-invasive, and potentially clinically relevant method to measure inhibitory control using the antisaccade task (Shafiq-Antonacci et al., 2003; Crawford et al., 2005, 2017; Antoniades et al., 2013). In the antisaccade task, participants are instructed to suppress a reactive eye movement (prosaccade) to a sudden onset of a laterally presented visual stimulus, to execute a voluntary eye movement (antisaccade) to a point in the visual field opposite the target (Hallett, 1978; Ramat et al., 2007). It is generally assumed (Peltsch et al., 2011) that reduced ability to inhibit the prepotent saccade typically results in slower responses or higher incorrectness in the antisaccade task (Sweeney et al., 2001; Butler and Zacks, 2006), which has been repeatedly found in older participants as compared with younger controls (Klein et al., 2000; Sweeney et al., 2001; Bojko et al., 2004; Abel and Douglas, 2007). However, these studies mainly focused on average reaction times and error rates when evaluating participant’s task performance and overlooked different sources of a worse performance of older participants as compared with younger controls during the antisaccade task (Reuter et al., 2005; Wiecki and Frank, 2013). Therefore, we reported full reaction time and error rate distributions and additional measures, like peak saccadic velocity and the saccade gain, as proposed in the internationally standardized antisaccade protocol (Antoniades et al., 2013).

Additionally, we used a probabilistic computational model to study the antisaccade task, referred to as the Stochastic Early Reaction, Inhibition, and Late Action (SERIA) model (Aponte et al., 2017), which links the concept of competing early processes (Logan et al., 1984; Camalier et al., 2007) with two voluntary actions that generate late prosaccade and antisaccade. This formal probabilistic approach enabled us to analyze the metrics not detectable by error rates and reaction time measures, especially inhibition failures, which are fast, reflexive prosaccades, which would be correct on prosaccade trials and errors on antisaccade trials (Aponte et al., 2019).

Moreover, previous studies typically conducted cross-sectionally antisaccade study design (Abel and Douglas, 2007; Peltsch et al., 2011) and thus it remains unknown whether antisaccade task metrics provide reliable estimate over time of inhibitory control for individual subjects, a prerequisite to qualify for clinically relevant markers of cognitive impairment. In order to bridge this gap, we further evaluated the test–retest reliability across two testing sessions per participant one week apart. In reference to our design analysis (reported in Materials and Methods), a total of 156 healthy participants (based on our power analysis) from two age groups (i.e., 78 young adults, age range: 20–35 years; 78 older adults, age range: 60–80 years) took part in a test–retest experimental design.

Based on the literature and our pilot study (see Materials and Methods, Pilot data), we hypothesized the following:

1. Significantly higher average error rates for older as compared to younger adults in the antisaccade task.
2. Longer saccadic reaction times for older adults as compared to younger adults in the antisaccade task.
3. High test–retest reliability [for reaction times, peak saccade velocity and gain indicating excellent or good reliability, i.e., intraclass correlation coefficient (ICC) > 0.6; McGraw and Wong, 1996].
4. Based on the SERIA model by Aponte et al., 2019, we expected significantly more inhibition failures for older adults as compared to young adults. Inhibition failures were classified as fast, reflexive prosaccades on prossacade trials and errors on antisaccade trials.

## Materials and Methods

### Dataset description

The data used in this study was recorded in our laboratory in the context of a larger project that aims to quantify age-effects on eye movement behavior and electroencephalography (EEG) recordings of resting-state and task-related activity. A total of 200 subjects [the first 44 subjects are considered pilot subjects (see below, Pilot data), the remaining 156 subjects were used for the main analysis, and these data have not been observed before the “in principal acceptance” of this Registered Report]. Two age groups (i.e., 100 young adults, age range: 20–35 years; 100 older adults, age range 60–80) took part in a test–retest experimental design, in which the same data recordings were performed one week apart (at the same time of day). Each recording included a test battery of seven experimental paradigms assessing key cognitive functions affected by age, such as visual perception, attention, working memory, episodic memory, cognitive control, and processing speed (Kozak and Cuthbert, 2016). For the purpose of this study, we focused on the eye-tracking data from the antisaccade task. This study was conducted according to the principles expressed in the Declaration of Helsinki. The study was approved by the Institutional Review Board of Canton Zurich (BASEC-Nr. 2017-00226). All participants gave their written informed consent before participation in the study and received a monetary compensation (the local currency equivalent of 25 United Stated Dollars).

For exploratory analysis, hypothesis generation and technical validation of our data processing pipeline, we conducted an analysis of a pilot dataset (described below, Pilot data). To further increase the transparency of our planned analyses, all processing scripts and data collected from our ongoing study can be found online in an OSF repository https://osf.io/4fu6r/.

### Power analysis

In order to estimate the sample size needed in our study, we performed a literature search and found 10 studies that compared antisaccade task performance between young and older adults (Olincy et al., 1997; Butler et al., 1999; Klein et al., 2000; Nieuwenhuis et al., 2000; Sweeney et al., 2001; Bojko et al., 2004; Eenshuistra et al., 2004; Bialystok et al., 2006; Raemaekers et al., 2006; Butler and Zacks, 2006; Olk and Kingstone, 2009).

Because none of the identified studies reported effects sizes, we estimated effect size for each study using reported mean reaction times and SDs, *F* values, and correlation values using the esc package for RStudio (Lipsey and Wilson, 2001). The average Cohen’s *d* effect size was 1.35, credibility interval (CI) [1.0511; 1.6527], and the effect size for our pilot study was equal to Cohen’s *d* = 0.77. To conduct a Bayesian meta-analysis, we used the R package metaBMA (Heck et al., 2017). Since publication bias overinflates published estimates of effect sizes (Ioannidis, 2005; Franco et al., 2016), we based our power analysis on the lowest estimate of the effect size for the differences in reaction time between young and old group (δ = 0.6). Considering that the data to be used in this study is was recorded in our laboratory in the context of a larger project with a fixed number of participants (see above, Dataset description), we used the simulation-based approach analysis design from (Schönbrodt and Wagenmakers, 2018) using the BFDA package (Schönbrodt and Wagenmakers, 2018). In our case, assuming an effect of δ = 0.6 and sample size equal to *n* = 156, simulation results showed that 0.5% of all simulated studies point toward the null hypothesis which specified the absence of an effect, that is, H0 of δ = 0 (the rate of false negative evidence). Conversely, 92% of simulated studies show support in favor of true positive results (H1 of δ > 0.6). The remaining 7.5% of simulated studies yielded inconclusive evidence. Evidence thresholds were defined at lower bound 1/6 and upper bound 6 (as proposed in the guidelines for the BFDA package; Schönbrodt and Wagenmakers, 2018).

### Sample description: inclusion and exclusion criteria

Inclusion criteria for participation in the study were left and right handedness, healthy male and female participants, with an age between 20 and 35 years (young participants) and 60–80 (old participants). Exclusion criteria for participation were as following: suffering from psychiatric symptoms, severe neurologic disorders (like epilepsy) or prior head injuries, a stroke, a transient circulatory disorder of the brain, diagnosis of dementia (Mini-Mental State Examination score <26), Huntington’s disease (HD), Parkinson’s disease, sensory and/or motor problems that interfere with computer tasks (e.g., the operation of a mouse), current use of psychotropic drugs (such as antidepressants, α-agonists, neuroleptics, mood stabilizers), intake of recreational synthetic or natural drug. Furthermore, data recorded from participants of the study was excluded from the analysis if the following criteria were met: incomplete data (i.e., missing data recording from the second session), eye tracker calibration failure, i.e., more than one visual degrees deviation on average across nine random visual stimulus presentations, <50% correct responses overall, >50% of trials rejected (for trial exclusion criteria, see below, Output measures).

### Experimental procedure and data acquisition

The experiment took place in a sound-attenuated and darkened room. The participant was seated at a distance of 68 cm from a 24-inch monitor (ASUS ROG, Swift PG248Q, display dimensions 531 × 299 mm, resolution 800 × 600 pixels resulting in a display: 400 × 298.9 mm, vertical refresh rate of 100 Hz). Participants completed the tasks sitting alone, while research assistants monitored their progress in the adjoining room. An infrared video-based eye tracker (EyeLink 1000 Plus, SR Research; http://www.sr-research.com/) positioned next to the monitor was used to record eye position at a sampling rate of 500 Hz and an instrumental spatial resolution of 0.01°. A stable head position of the participant was ensured via a chin rest and via experimenter’s instruction to stay as still as possible during data recordings. Moreover, for higher precision of the calibration and validation results, we used a small target sticker placed on the participant’s forehead, which allowed head movement compensation even during blinks. The eye tracker was calibrated and validated with a 9-point grid before each experimental block. In a validation step, the calibration was repeated until the average error for all points was be <1°. The eye-tracking device was recalibrated after every experimental block of the experiment (consisting of either 60 prosaccade trials or 40 antisaccade trials, see below).

**Figure 1.**
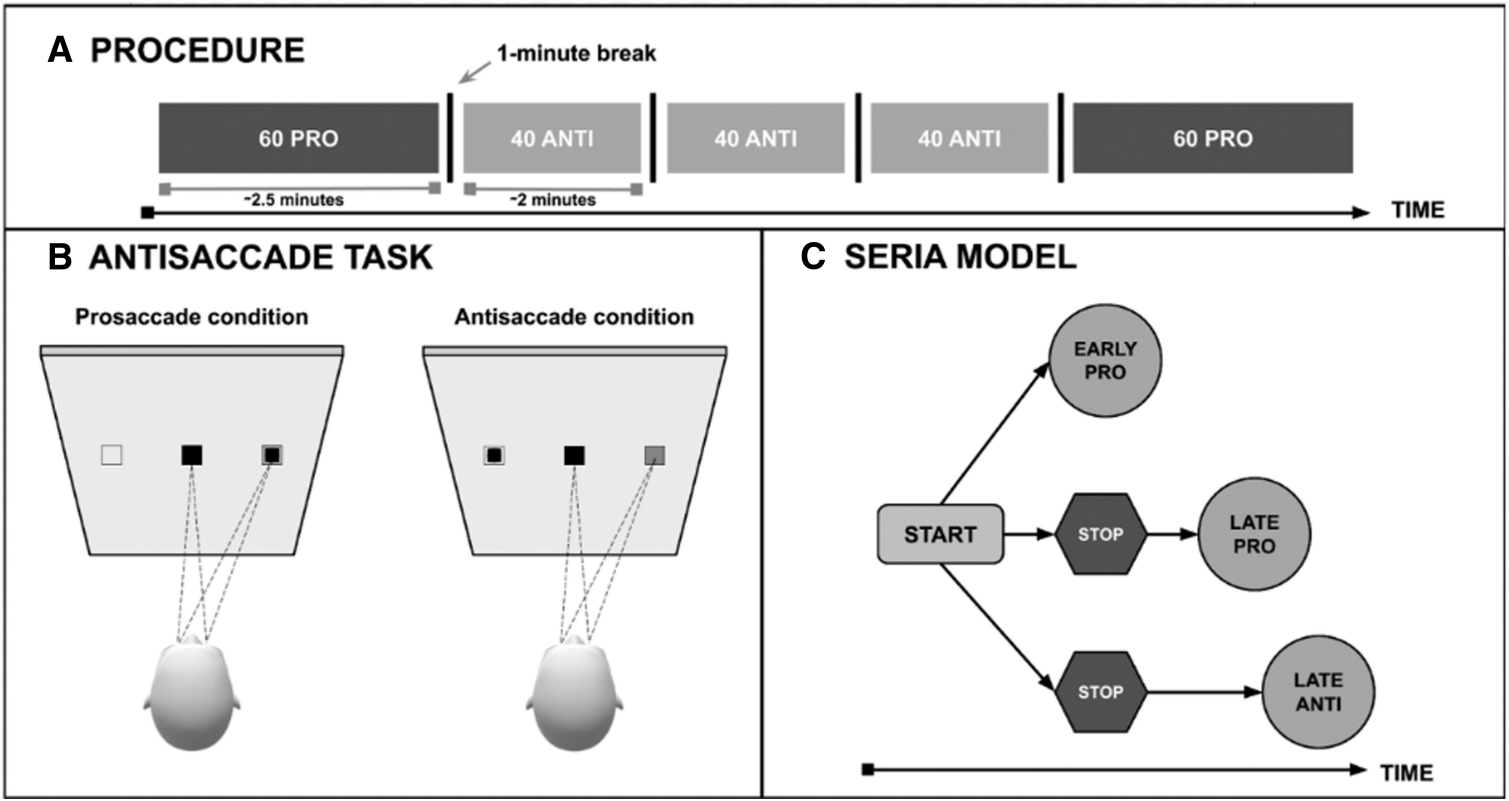
***A***, The experimental procedure of a single run, consisting of prosaccade task (PRO) and antisaccade task (ANTI) blocks, which each consisted of either 40 or 60 trials per block. There was 1 min between each block. ***B***, Schematic top view of the experimental setup and gaze behavior during a prosaccade and antisaccade condition trial. The black square represents the target fixation in the center of the screen, and the smaller black square represents the peripheral stimulus (cue). The peripheral stimulus is presented 1000 ms on the screen and starts after a duration of the target fixation of 800–1200 ms. ***C***, The sequence of latent events assumed by the SERIA model, generating as output either early prosaccades (EARLY PRO), late prosaccades (LATE PRO), or antisaccade events (LATE ANTI).

The experiment was programmed in MATLAB 2016b, using the PsychToolbox extensions (Brainard, 1997; Pelli, 1997). The experimental stimuli were based on an internationally standardized protocol for antisaccade testing, allowing comparisons between different labs and clinics (Antoniades et al., 2013). Visual stimuli consisted of horizontally arranged stimuli, targets presented on the screen were of a high contrast ratio (i.e., 11.05) to minimize issues related to light-adaptation level. Each trial started with a central fixation square (visual angle of 0.6319°). Subsequently, a black square (visual angle of 0.6319°) was presented on a gray background for 1000 ms. To avoid excessive head movements (John Leigh and Zee, 2006), stimuli were always presented at the same vertical height and offset from the center (with an amplitude of 10° from the screen center). In prosaccade trials participants were instructed to perform a saccade to the peripheral stimulus, the black square presented laterally, and in antisaccade trials to perform a saccade to a corresponding location at the opposite side of the screen. The next trial started 1000–3500 ms after the target fixations of the prosaccade or antisaccade. Stimuli were presented in equal numbers to the left and right side of the screen (20 per visual hemifield in the antisaccade condition and 30 per visual hemifield in the control, prosaccade condition). In each experimental trial, the location (left or right) of the peripheral stimulus is randomly assigned. The standardized test protocol (Antoniades et al., 2013) consisted of three blocks for the antisaccade task (40 trials per block) and two blocks of the prosaccade task (60 trials per block, control task; Fig. 1*A*), presented in prosaccade-antisaccade-antisaccade-antisaccade-prosaccade order to account for time-dependent effects. Before the first prosaccade block, 10 practice trials, and before the first antisaccade block, five practice trials were presented. Practice trials were aimed to acquaint the participant with our experimental procedures and were not statistically analyzed.

Each participant completed two recording sessions in a test–retest experimental design with an interval of one week (acceptable range: 7–9 d) between recording sessions (at the same time of day). During both visits, the same experimental protocol was followed, including the same order of tasks.

### Eye-tracking data preprocessing

The EyeLink 1000 tracker computed eye-position data, measures pupil diameter and identified events such as saccades, fixations, and blinks. Saccade onsets were detected using the eye tracking software default settings: acceleration larger than 8000°/s^2^, a velocity above 30°/s, and a deflection above 0.1°. We extracted the following information about the saccades: start and end time, duration, coordinates of start positions and end positions on the computer screen in pixels, amplitudes, and eye velocity.

Fixations were defined as time periods without saccades and eye blinks were regarded as a special case of a fixation, where the pupil diameter was either zero or outside a dynamically computed valid pupil. Thus, fixation might include small saccades (i.e., microsaccades), which fall below the threshold for saccade detection. In the present study, we focused only on standard saccades (not microsaccades). Consequently, all considered output measures were based on these standard saccades.

### Output measures

The output measures of interests were: reaction time for the first saccade, defined as time from onset of the peripheral stimulus to the start of the saccade (Antoniades et al., 2013), regardless of whether the saccade was elicited in the correct direction. An error was defined as a saccade toward the stimulus in an antisaccade block, and away from the stimulus in a prosaccade block. The error rate for each participant was calculated as the proportion of erroneous trials to all valid trials separately for antisaccade and prosaccade blocks. Additionally, we extracted the peak saccadic velocity for each saccade as provided by eye tracker recordings. The gain of the first saccade was calculated as a ratio of actual saccade amplitude divided by the desired saccade amplitude (in our experimental setup equal to 10°, based on Antoniades et al., 2013). Trial exclusion criteria were based on Antoniades et al. (2013): occurrences of eye blinks between the cue presentation and the saccade, reaction times of <50-ms duration, a saccade onset later than 800 ms after cue presentation. If 50% or more trials were rejected, the subject was excluded.

### Data analysis

The two primary goals of our study were testing the presence of age differences in all outcome measures and inspecting their reliability across the two test–retest recording sessions. For each of the goals, we described below the analysis pipeline, including all preprocessing steps and planned analyses.

### Age differences

The presence of age differences in all outcome measures [reaction times, error rates, peak saccadic velocities, saccade gains, model parameters of PRO-Stop-Antisaccade (PROSA) and SERIA: inhibitory fail probability and inhibitory fail reaction time (for description of model parameters, see below, Computational model)] was investigated. Single trials that were not excluded during preprocessing (for trial exclusion criteria, see above, Output measures) from all subjects were used for fitting a multivariate Bayesian generalized linear mixed model. We used the brms package which offers robust estimates in the context of multilevel modeling (Kozak and Cuthbert, 2016; Bürkner, 2017, 2018). To improve convergence and guard against overfitting, we used weakly informative Cauchy priors in line with the recommendations for Bayesian regression models (Gelman et al., 2008). We used the data from both time points and random intercepts were added for the participant factor. The predictor type (levels: antisaccade condition, prosaccade condition) was included to account for the influence of the type of the experimental block as shown in Equation 1:

(1) $$\left[dv^{\prime }s\right]∼age_{group}*type+(1|participantID).$$

The model fitted at the same time the four dependent variables (reaction times, error rates, peak saccadic velocities, saccade gains). To account for possible multiple comparisons, we corrected the effective number of tests using the approach of Nyholt (2004), which, based on the ratio of observed eigenvalue variance to its maximum, gives the proportional reduction in the number of variables in a set, and therefore provides a useful alternative to more computationally intensive permutation tests. Then, we reported the adjusted α level of the Bayesian posterior CIs.

### Test–Retest Reliability

In order to quantify test–retest reliability for the output measures collected at the two recording sessions per subject, we calculated one-way random effects model ICCs using the absolute agreement measure among multiple observations (Bhapkar, 1966; Finn, 1970; McGraw and Wong, 1996), with the open source software package irr (https://CRAN.R-project.org/package=irr) for reaction times, peak saccade velocity, error rates and gain of the first saccade, and the quantities obtained from the computational model. We used the following, generally adopted interpretation of ICC, introduced by Cicchetti (1994): <0.40 (poor reliability), between 0.40 and 0.59 (fair reliability), between 0.60 and 0.74 (good reliability), and between 0.75 and 1.00 (excellent reliability).

Additionally, we also used Bland–Altman plots (Bland and Altman, 1999) for graphical comparison of two measurements from test and retest recording sessions. In the Bland–Altman plot, each sample is represented on the graph by plotting the mean value of the two assessments against the difference value between them. The chart can then highlight possible anomalies, such as revealing that one time point overestimates high values and underestimates low values (Kalra, 2017). We also used a quantitative method assessing the agreement of test and retest (first and second measurement). It is based on a priori defined limits of agreement (as for other relevant measures, it was recommended that 95% of the data points should lie within ±1.96 SD of the mean difference–limits of agreement; Sedgwick, 2013; Earthman, 2015).

### Computational model

We used the PROSA and the SERIA model (Aponte et al., 2017) to fit experimental data from the antisaccade task to estimate latent, not directly observable processes. PROSA and SERIA are inspired by the hypothesis that antisaccades are the result of competing decision mechanisms that interact nonlinearly with each other. This approach is based on previous proposals and fits the to-be explained reaction time and error rate in the double step and search step tasks (Noorani and Carpenter, 2013). SERIA and PROSA offer a formal, probabilistic approach to the antisaccade task and provide detailed information about the participants’ performance.

Briefly, the PROSA model assumes that the reaction time and the response (either prosaccade or antisaccade) in a given trial are caused by the interaction of three competing processes: eliciting a prosaccade, inhibitory command to stop a prosaccade, and eliciting an antisaccade. On the other hand, in the SERIA model, four different units can be distinguished: the early prosaccade unit, the inhibitory unit (that can stop early prosaccades), the antisaccade unit, and the late prosaccade unit (for an illustration of the model, see Fig. 1*C*). The exact details of The PROSA and SERIA are described in Aponte et al. (2017). We used the SEM toolbox (Aponte et al., 2017) and the method for model fitting used by Aponte et al. (2017), based on the Metropolis–Hastings algorithm (Gelman et al., 2003). Moreover, we applied a hierarchical method of fitting the model, which treats the group mean as before the parameters and therefore offers a form of regularization based on observations from the population. Our data (only valid trials, see above, Sample description: inclusion and exclusion criteria) were entered into the models as a structure with fields representing the reaction time and the corresponding action (either prosaccade or antisaccade). The result was an array of samples from the target distribution, which was used to compute summary statistics. To investigate whether the behavior of young and elderly adults is better explained by PROSA or SERIA model, we compared the PROSA and SERIA model fits for young and the old participants, based on obtained model evidence, as described previously (Aponte et al., 2017).

## Pilot data

The primary purpose of the pilot data analysis was to assure that our test–retest experimental design is a stable and reliable method to further testing age differences. According to our power analysis (see Materials and Methods), the pilot dataset is underpowered, and thus, we did not conduct any statistical tests on it. Instead, we present the raw distributions and reciprobit plots of reaction times. Additionally, we include ICCs for four output measures and Bland–Altman plots for reaction times and error rates, which need to be interpreted with caution, because of the small sample size (methods for obtaining them are described in Materials and Methods, Test–Retest Reliability).

### Participants

Data for the pilot study were recorded from 22 healthy young subjects (20–25 years, mean age 23.6 years, SD = 3.3 years) and 22 healthy older subjects (>60 years, mean age 68.9 years, SD = 2.9 years). Data from four participants were discarded because of low performance in the antisaccade task (error rate >50%). The final sample used for pilot data analysis thus consists of 40 participants.

### Results

#### Output measures

Across all 40 subjects, a total of 19,200 trials were recorded, from which 906 trials were excluded based on the trial exclusion criteria described in Materials and Methods. Out of the total 906 excluded trials, 288 were occurrences of eye blinks between the cue presentation and the saccade, 526 had reaction times of <50-ms duration, and 92 had a saccade onset later than 800 ms after cue presentation. For each experimental trial, we extracted the following: reaction time for the first saccade, information if the participant looked in the correct direction or not, peak saccadic velocity, gain of the first saccade. Table 1 illustrates the results obtained from the pilot dataset. Descriptives of each of the extracted measures are presented separately for prosaccade and antisaccades, young and old participants.

**Table 1:** Descriptives of reaction times for the first saccade, error rate, gain of the first saccade (ratio of actual saccade amplitude divided by the desired saccade amplitude), and peak saccadic velocity for the prosaccade and antisaccade condition for the young and old group

	Young group (*n* = 20)								Old group (*n* = 20)							
	Prossacade condition				Antisaccade condition				Prossacade condition				Antisaccade condition			
	Mean	SD	Min	Max	Mean	SD	Min	Max	Mean	SD	Min	Max	Mean	SD	Min	Max
Reaction time (ms)	268	83	51	790	303	88	51	786	309	118	51	796	360	130	51	794
Error rate (%)	1.3	1.92	0	10	7.83	6.54	0	27.5	5.35	5.31	0	21.6	17.2	14.7	0	57.4
Gain of the saccade (ratio)	0.81	0.18	0.01	2.58	0.79	0.22	0.01	3.48	0.76	0.28	0.01	3.07	0.7	0.32	0.01	4.03
Peak saccadic velocity (°/s)	331	229	45	3270	326	259	5.0	3270	288	193	44.0	3270	267	210	44	3270

**Figure 2.**
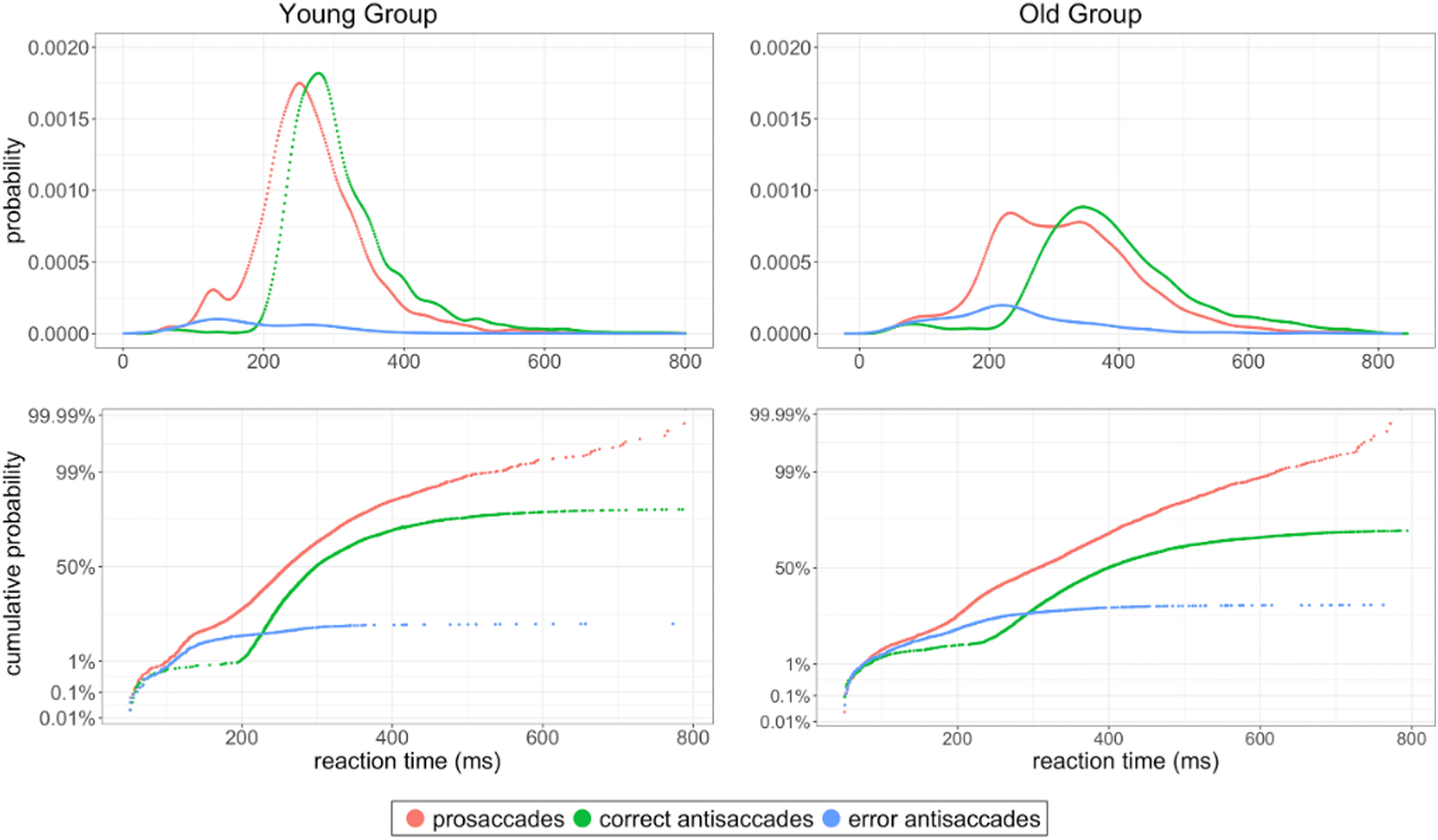
Top panels, Raw distributions with error responses plotted as a cumulative proportion of the total number of trials for young and old group, showing a rightward shift of the correct antisaccade distribution relative to both the prosaccade and error antisaccades distributions. Bottom panels, The same data as shown above as reciprobit plots. Error responses are plotted as a cumulative proportion of the total number of trials.

To assess the contribution of different factors to an experiment’s results (Carpenter et al., 2007; Noorani and Carpenter, 2013), we used reciprobit plots, as recommended in the internationally standardized antisaccade protocol (Antoniades et al., 2013). Figure 2 shows data distributions of all trials from the young group (Fig. 2, left) and the old group (Fig. 2, right). In the antisaccade task, the latency distributions of correct antisaccades and error prosaccades have characteristics that are different from those seen in the control (prosaccade) condition. The error responses were slightly delayed for the antisaccade as compared with the prosaccade condition (especially evident in the old participants), and it is visible that there were far fewer errors for prosaccades than for antisaccades.

#### Test–retest reliability

**Figure 3.**
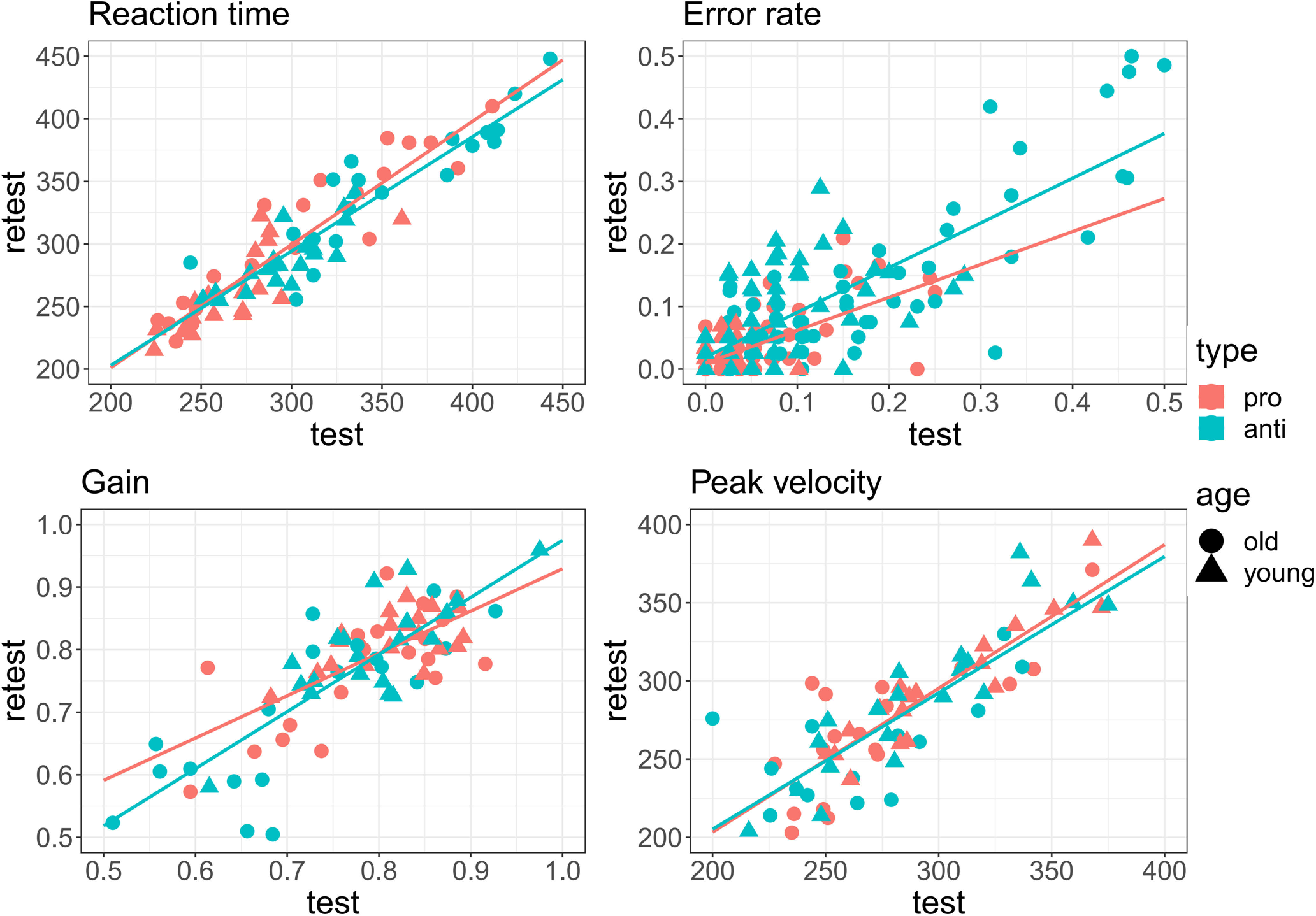
Paired distributions of four output measures (reaction time, error rate, gain, peak velocity) for test and retest measurement timepoints. Each point represents one subject. Solid red and blue lines correspond to linear regression model fit for prosaccades and antisaccades, respectively.

Our pilot study confirmed the high test–retest reliability for reaction times, first saccade gains and peak saccadic velocity (see Table 2). A possible explanation for the low ICCs for error rates of young participants might be that error rates, especially for the prosaccade task are low (<5% of all trials), and thus, we had not enough data to obtain stable estimates for this output measure. Figure 3 displays distributions of four output measures (reaction time, error rate, gain, peak velocity) for test and retest measurement timepoints.

**Table 2:** ICCs with 95% confidence intervals in brackets for four output measures, separately for prosaccade and antisaccade condition and for old and young group

	Young group (*n* = 20)		Old group (*n* = 20)	
	Prosaccades	Antisaccades	Prosaccades	Antisaccades
Reaction time	0.66 (0.51; 0.77)	0.64 (0.53; 0.71)	0.85 (0.78; 0.9)	0.8 (0.74; 0.85)
Error rate	0.22 (0.09; 0.41)	0.45 (0.33; 0.56)	0.47 (0.27; 0.62)	0.75 (0.67; 0.86)
Gain of the saccade	0.51 (0.32; 0.65)	0.62 (0.52; 0.7)	0.64 (0.49; 0.75)	0.61 (0.5; 0.7)
Peak saccadic velocity	0.51 (0.33; 0.66)	0.5 (0.39; 0.61)	0.71 (0.58; 0.8)	0.59 (0.48; 0.69)

**Figure 4.**
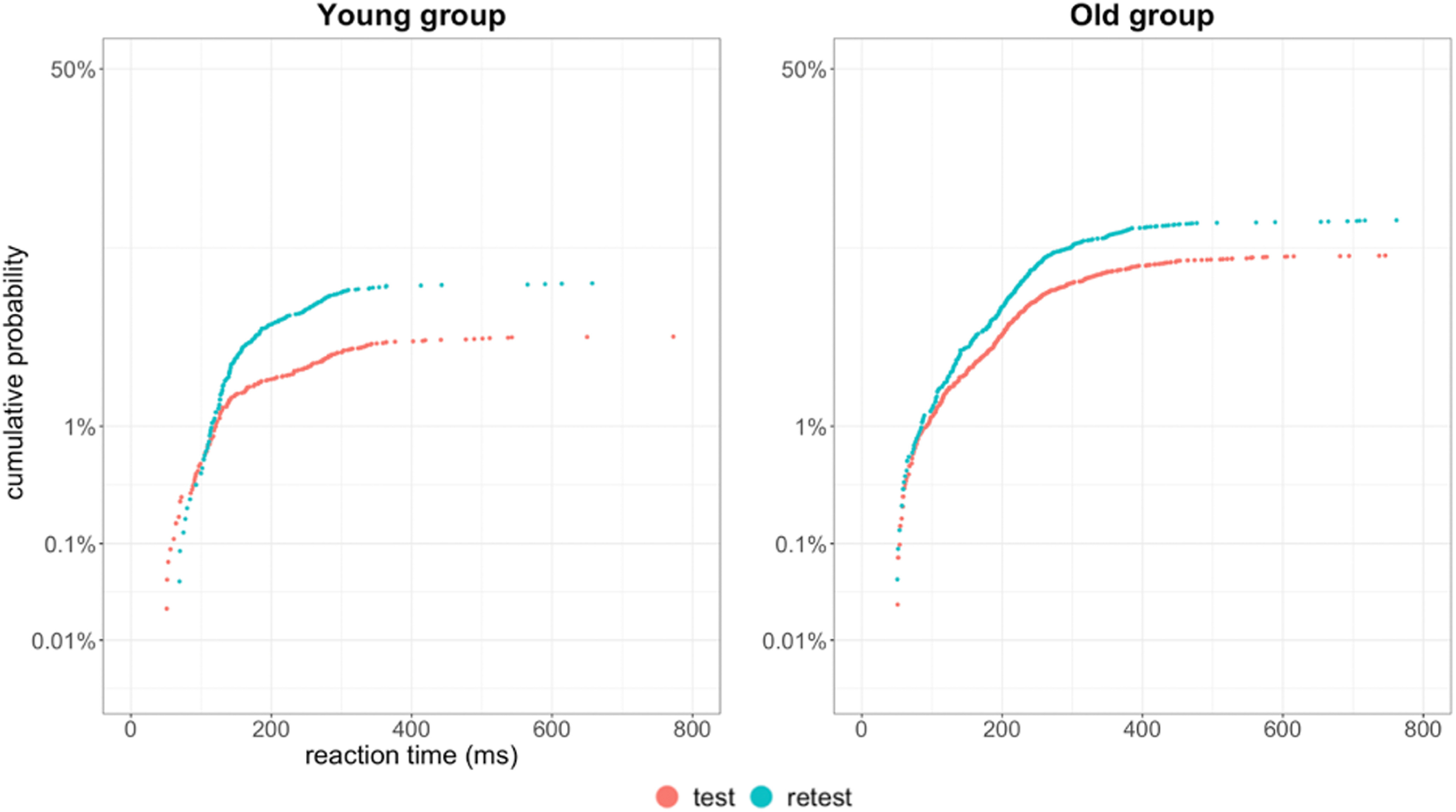
Reciprobit plots for error rate in the antisaccade trials, comparison for the young and old group, for test and retest. Error responses are plotted as a cumulative proportion of the total number of trials.

Additionally, Bland–Altman plots were used to graphically represent the agreement between the two measurements. According to Kalra (2017), 95% of the data points should lie within ±1.96 SD of the mean difference limits of agreement. From the data in Figure 4, 5 it is apparent that our study design can provide reliable results and is suitable for further testing in the main study, with a larger sample size.

**Figure 5.**
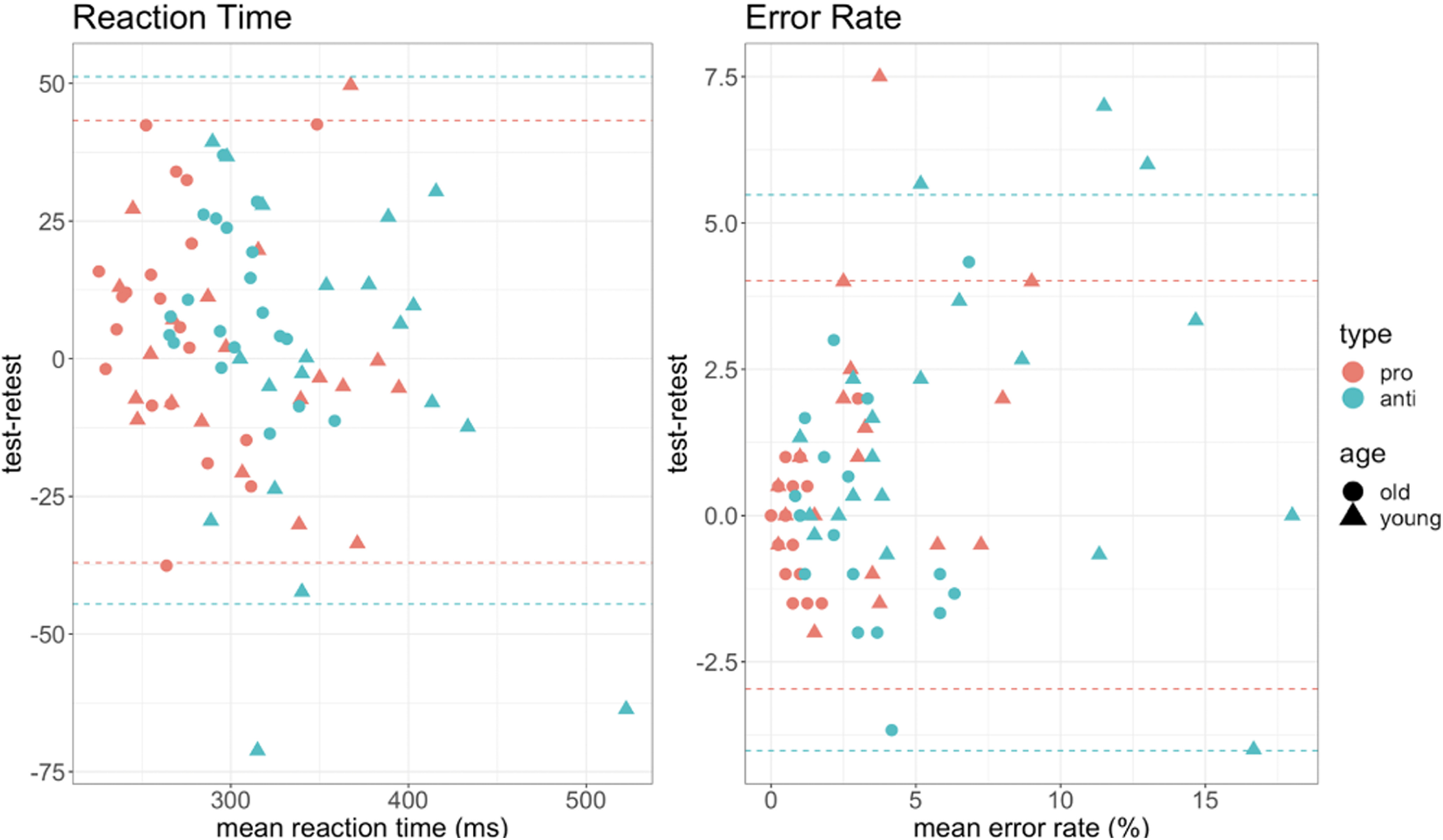
Bland–Altman plots for two measures of interest; error rate and reaction time. Horizontal dashed lines are drawn at the limits of agreement, which are defined as the mean difference plus and minus 1.96 times the SD of the differences.

## Stage 2

### 

#### Sample description

Two age groups (i.e., 78 young adults: age range: 20–35 years; 78 older adults: age range 60–80, 74 women) took part in a test–retest experimental design, in which the same data recordings were performed one week apart (at the same time of day).

Of all 156 participants, seven were excluded from the old group and five from the young group according to the participants’ exclusion criteria described in Materials and Methods, leaving a sample of 144 participants.

A total of 72,960 trials were recorded in both sessions together. Of these, a total of 3754 trials were excluded: 709 were occurrences of eye blinks between the cue presentation and the saccade, 1891 had reaction times of <50-ms duration, and 1154 had reaction times longer than 800 ms after cue presentation.

#### Age effects

Age differences were investigated with a multivariate Bayesian generalized linear mixed model in all four outcome measures: reaction times, error rates, peak saccadic velocities, and saccade gains. Data from both time points were used, and random intercepts were added for the participants. Factor type (levels: antisaccade condition, prosaccade condition) was included to account for the influence of the type of experimental block. The multivariate model with a dependent variable for each of the outcome measures provided the estimates summarized in Table 3. To account for multiple comparisons, we corrected the effective number of tests using Nyholt’s (2004) approach. The effective number of variables was calculated (3.86), and after the correction for multiple comparisons, the adjusted α level of the Bayesian posterior Credible Interval (CIs) was equal to 1.3%, and thus, the model estimates are presented for a CI of 98.7%.

**Table 3:** Bayesian model estimates

Dependent variable	Parameter	Estimate (error)	CI_lower_	CI_upper_
Reaction time	Age Type Age:type	32.94 (6.75) –43.86 (1.93) –3.37 (2.65)	16.06 –48.68 –10.37	49.85 –38.99 3.04
Error rate	Age Type Age:type	0.06 (0.01) –0.07 (0.00) –0.04 (0.01)	0.04 –0.08 –0.05	0.09 –0.06 –0.02
Peak velocity	Age Type Age:type	–9.24 (9.16) 11.65 (3.38) 0.31 (4.51)	–36.41 3.08 –11.03	10.40 20.08 11.73
Gain	Age Type Age:type	–0.07 (0.01) 0.02 (0.00) 0.02 (0.01)	–0.09 0.01 0.01	–0.04 0.03 0.04

Younger group and antisaccade condition are references (i.e., older group had on average 32.94 ms longer reaction time). CI = 98.7% CI.

**Figure 6.**
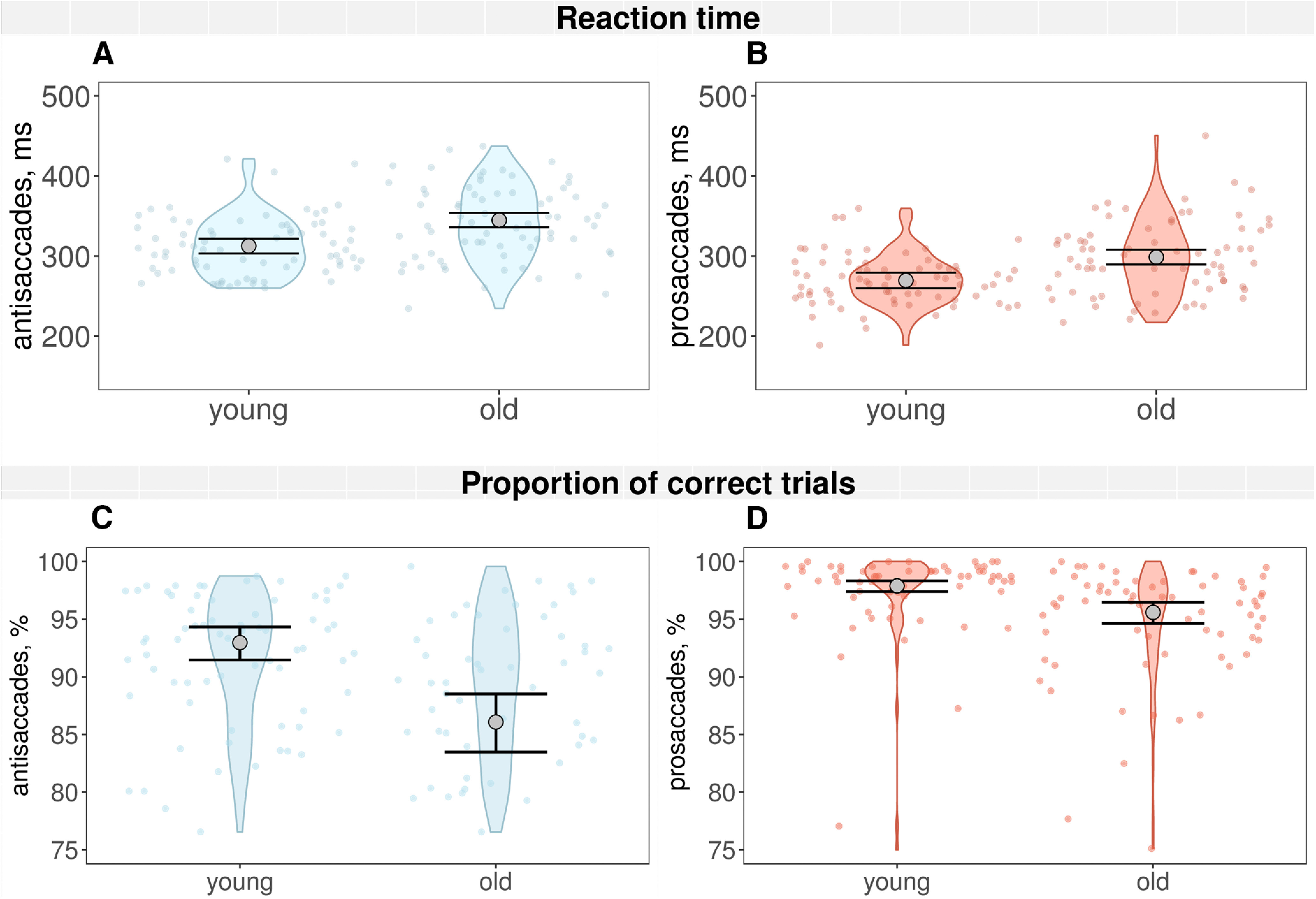
Reaction times (***A***, ***B***) and proportion of correct trials (***C***, ***D***); plots of the Bayesian model predictions. Large gray points show mean fitted values; the mean of posterior distribution and 98.7% CIs. Small red (prosaccades) and blue (antisaccades) dots represent means over all blocks (two for prosaccades, three for antisaccades) for all the participants.

In both conditions, older people committed significantly more errors than younger people, 6% (CI [4%,9%]), and had significantly longer reaction times (Fig. 6); the average difference between the two groups’ reaction times was 32.94 ms (98.7% CI [16.06,49.85]). Likewise, their gain was significantly smaller than young people’s. It is possible that peak velocity in the older group was marginally (9.24 CI [−36.41,10.40]) slower than in the younger group, but this difference was not statistically robust.

Compared with the antisaccade condition, the prosaccade had on average 43.86-ms shorter reaction times (CI [−46.68,−38.99]). We also found significant differences in the error rate: 7% (CI [6%,8%]) on average, in the peak saccadic velocity, prosaccades were faster by 11.65 ms (98.7% CI [3.08,20.08]), and in the gain of the first saccade, which was on average 0.02 higher than for the antisaccade condition (98.7% CI [0.01,0.03]). Moreover, we found significant interaction effects between the age of the participant and type of the condition for the error rate: 4% (98.7% CI [2%, 5%]), and the gain of the saccade: 0.02 (98.7% CI [0.01, 0.04]). All CIs are presented in Table 3 with estimated errors.

#### Test–retest reliability

The test–retest reliability of the output measures collected at the recording sessions was quantified with one-way random effects model ICCs.

**Figure 7.**
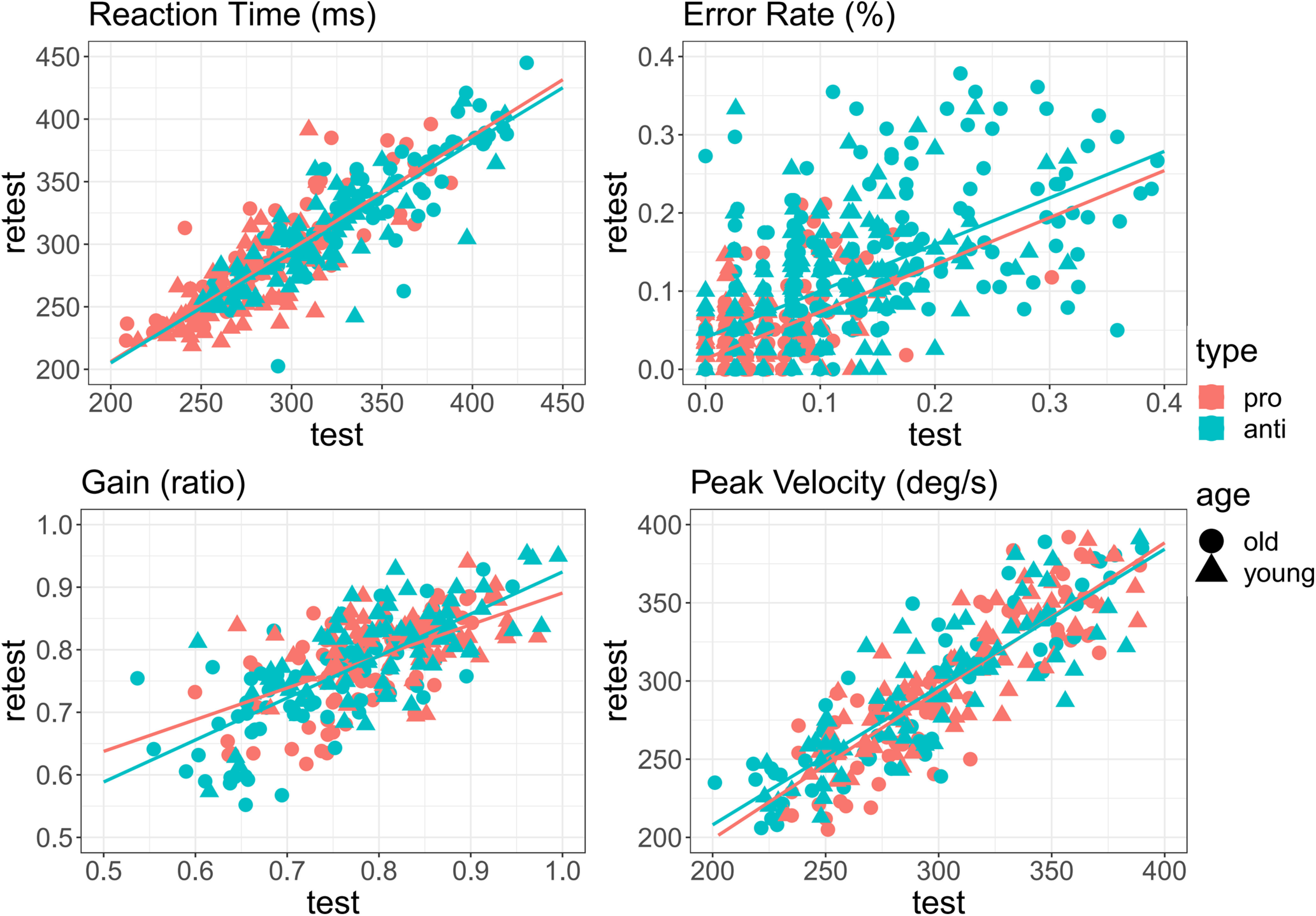
Paired distributions of four output measures (reaction time, error rate, gain, peak velocity) for test and retest measurement timepoints. Each point represents one subject. Solid red and blue lines correspond to linear regression model fit for prosaccades and antisaccades, respectively.

The reaction time and the error rate shown in Table 4 indicate that our study design can provide reliable results. Except for the prosaccade error rate for younger participants, all other ICCs resulted in excellent or good reliability (ICC > 0.6). Overall, we found higher ICCs for all four measures for the older group than for the younger group (Fig. 7).

**Table 4:** ICCs with 95% CIs in brackets for four output measures, separately for prosaccade and antisaccade condition and for older and younger groups

	Younger group (*n* = 73)		Older group (*n* = 71)	
	Prosaccades	Antisaccades	Prosaccades	Antisaccades
Reaction time	0.74 (0.61; 0.83)	0.75 (0.63; 0.84)	0.87 (0.80; 0.92)	0.89 (0.82; 0.93)
Error rate	0.52 (0.32; 0.69)	0.77 (0.65; 0.85)	0.70 (0.56; 0.80)	0.73 (0.59; 0.82)
Gain of the saccade	0.47 (0.291; 0.577)	0.66 (0.51; 0.77)	0.64 (0.47; 0.75)	0.89 (0.82; 0.93)
Peak saccadic velocity	0.52 (0.33; 0.68)	0.59 (0.41; 0.71)	0.59 (0.40;0.72)	0.89 (0.82; 0.93)

**Figure 8.**
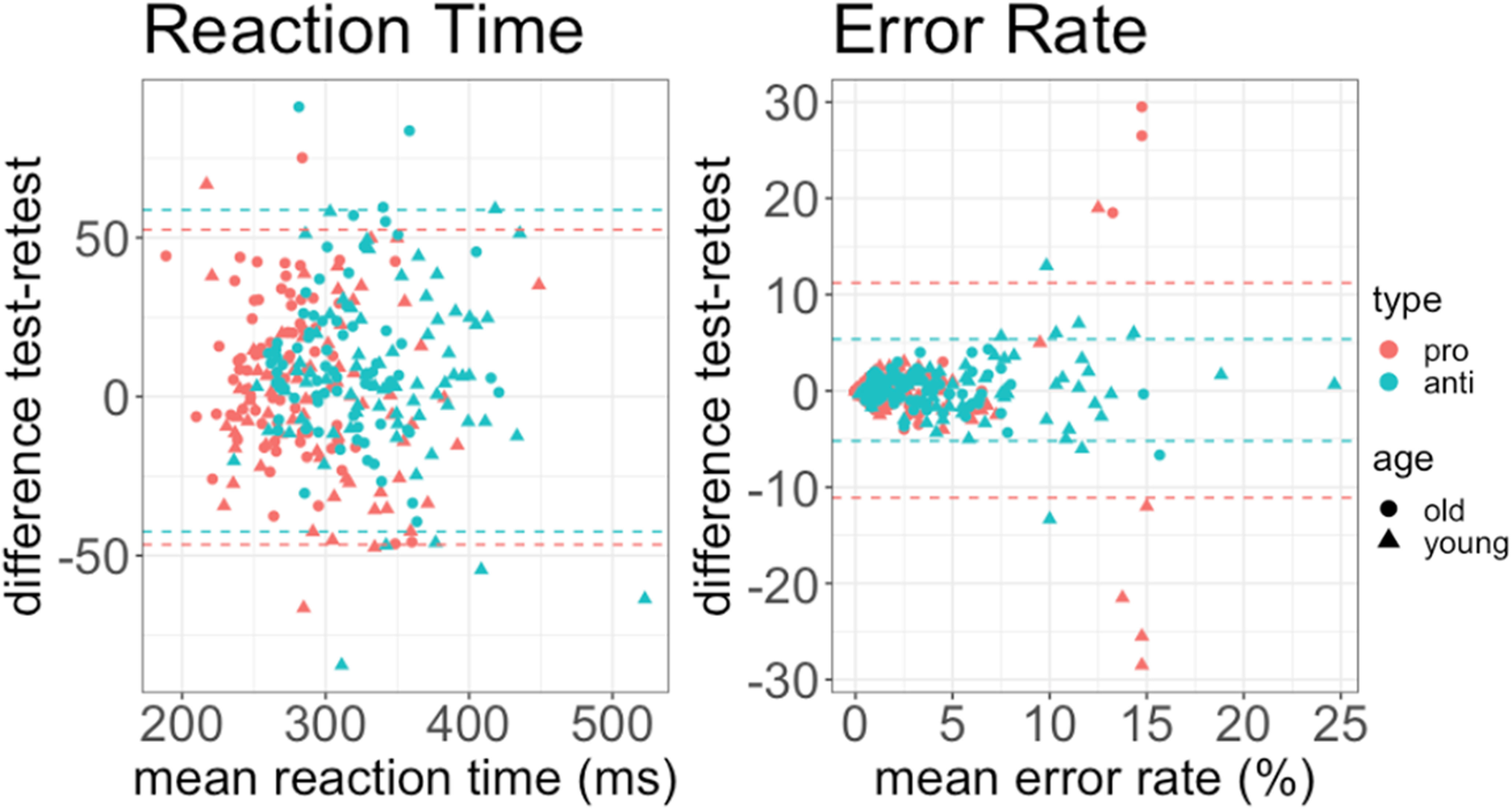
Bland–Altman plots for two measures of interest; reaction time and error rate. Horizontal dashed lines are drawn at the limits of agreement, which are defined as the mean difference plus and minus 1.96 times the SD of the differences.

Furthermore, we created Bland–Altman plots (Fig. 8) that graphically represent the agreement between the two measurements. Additionally, we calculated the percentage of points that lay within ±1.96 SD of the mean difference limits of agreement.

We obtained the following results for prosaccades: for reaction times, 97% of our data points lay within ±1.96 SD of the mean difference limits of agreement, and for the error rates, 94% of them. For antisaccades, 94% of data points for both reaction times and error rates lay within ±1.96 SD of the mean difference limits of agreement.

## Computational model

We used the PROSA and SERIA models to decompose the task into underlying latent components representing the reaction time and the corresponding action: either prosaccade or antisaccade. Additionally, we included an age factor in the output structure.

Two multivariate models were fitted. The main goal was to compare a latent variable, inhibition failure. The PROSA and SERIA models both classify inhibition failures as fast, reflexive prosaccades on prosaccade trials and errors on antisaccade trials.

## PROSA

For the PROSA model, we fitted a multivariate model with two dependent variables: inhibitory fail probability and an inhibitory fail reaction time. All estimates are provided in Table 5. To account for multiple comparisons, we corrected the effective number of tests using Nyholt’s (2004) approach, so the model estimates are presented for a CI of 96.9%.

**Table 5:** Bayesian model estimates for the PROSA model

Dependent variable	Parameter	Estimate (error)	CI_lower_	CI_upper_
Inhibitory fail probability	Age Type Age:type	0.08 (0.01) 0.87 (0.01) –0.11 (0.02)	0.06 0.85 –0.14	0.09 0.90 –0.08
Inhibitory fail reaction time	Age Type Age:type	0.19 (0.05) 0.69 (0.05) 0.11 (0.07)	0.07 0.59 –0.05	0.30 0.81 0.27

Younger group and antisaccade condition are references (i.e., older group had on average 8% higher probability for inhibitory failures). CI = 96.9% CI.

Compared with the young people, older adults committed significantly more inhibition failures: 8% (96.9% CI [6%,9%]). They also had significantly longer inhibitory failure reaction times: 19 ms (96.9% CI [7.00,30.03]). The short prosaccades were more commonly classified as inhibition failures than the late prosaccades, according to their definitions: reflexive prosaccades on prosaccade trials and errors on antisaccade trials (Aponte, 2017).

## SERIA

Given that the SERIA model includes one more unit than the PROSA model, late saccade, we also incorporated it in the Bayesian multivariate model. Crucially, late responses can trigger prosaccades and antisaccades with a specific probability (Aponte, 2017).

Finally, we fitted a multivariate model with four dependent variables: late saccade probability, late saccade reaction time, inhibitory fail probability, and inhibitory fail reaction time. All estimates are provided in Table 6. To account for multiple comparisons, we corrected the effective number of tests using Nyholt’s (2004) approach, so the model estimates are presented for a CI of 98.5%.

**Table 6:** Bayesian model estimates for the SERIA model

Dependent variable	Parameter	Estimate (error)	CI_lower_	CI_upper_
Late saccade probability	Age Type Age:type	0.05 (0.01) 0.92 (0.01) –0.08 (0.01)	0.03 0.90 –0.11	0.07 0.94 –0.05
Late saccade reaction time	Age Type Age:type	0.39 (0.05) 0.09 (0.06) –0.15 (0.08)	0.25 –0.04 –0.34	0.52 0.23 0.05
Inhibitory fail probability	Age Type Age:type	0.03 (0.01) 0.08 (0.01) –0.08 (0.02)	0.01 0.05 –0.11	0.07 0.12 –0.05
Inhibitory fail reaction time	Age Type Age:type	0.08 (0.04) 0.29 (0.04) –0.17 (0.06)	0.01 0.19 –0.30	0.18 0.39 –0.03

Younger group and antisaccade condition are references (i.e., older group had on average 5% higher probability for late saccades). CI = 98.5% CI.

As expected, the SERIA model predicted significantly more inhibition failures for older adults than for young adults: 3% (98.5% CI [1%, 7%]).

Moreover, compared with young people, older adults have significantly longer inhibitory fail reaction times: 8 ms (98.5% CI [1.00, 18.00]). Again, prosaccades were more commonly classified as inhibition failures: 8% (98.5% CI [5, 12]). Furthermore, compared with the young people, older adults had significantly longer late saccade reaction times: 39 ms (98.5% CI [25.00,52.00] on average, and significantly higher probability for late saccades: 5% (98.5% CI [3%, 7%]).

Finally, we investigated which model explains our data better. A Bayesian modeling approach was used along with the method for model fitting (described in detail in Aponte, 2017) based on the Metropolis–Hastings algorithm (Gelman et al., 2003). This approach allowed us to compare PROSA and SERIA models for younger and older groups based on their evidence. Models were scored using their log marginal likelihood.

We applied a hierarchical method of fitting the model; this model treats the group mean as before the parameters and therefore offers a form of regularization based on observations from the population.

The SERIA model had higher evidence than the PROSA model (*ΔLME *>* *3000) for both age groups. Both SERIA and PROSA provided higher evidence for the younger group: for SERIA, *ΔLME *>* *8200; for PROSA, *ΔLME *>* *8890.

### Exploratory analysis

#### Reliability of the SERIA model

Although not a primary goal of our study, we considered the reliability of measures obtained from the SERIA model as crucial information. Age differences in the model parameters are only meaningful if reliability is given. Thus, we have further investigated the ICCs for the four latent measures from the SERIA mode. The ICCs for the model parameters in the antisaccade task exhibited fair reliability (ICC > 0.40) in both age groups. Only inhibitory fail reaction time for the older group displayed low reliability (ICC = 0.31). In the prosaccade task, all measures except the late prosaccade reaction time only achieved poor reliability. All ICCs with the estimated errors and 95% CI for ICC population values are presented in Table 7.

**Table 7:** ICCs with 95% CIs in brackets for four output measures of SERIA model separately for prosaccade and antisaccade conditions and for older and younger groups

	Younger group (*n* = 73)		Older group (*n* = 71)	
	Prosaccades	Antisaccades	Prosaccades	Antisaccades
Inhibitory fail probability	0.36 (0.15; 0.54)	0.78 (0.67; 0.83)	0.16 (0.01; 0.36)	0.81 (0.71; 0.88)
Inhibitory fail reaction time	0.06 (0.00; 0.29)	0.42 (0.2; 0.59)	0.22 (0.00; 0.44)	0.31 (0.08; 0.51)
Late prosaccade probability	0.04 (–0.19; 0.27)	0.70 (0.56; 0.80)	0.20 (–0.03; 0.41)	0.53 (0.34; 0.68)
Late prosaccade reaction time	0.52 (0.16; 0.55)	0.52 (0.33; 0.68)	0.38 (0.16; 0.55)	0.86 (0.79; 0.91)

A potential confounding factor was the stability of the model over multiple repetitions. Thus, we have fitted the identical model to the data 100 times. As the SERIA model is probabilistic, the results are expected to vary across the repetitions. Our analyses demonstrated that the SERIA model provided satisfactory stability model parameters for our results Table 8 depicts the 2.5% and 97.5% quantile from each variable of the model.

**Table 8:** The 2.5% and 97.5% quantile from each variable of the SERIA model over 100 repetitions

	Younger group (*n* = 73)		Older group (*n* = 71)	
	Prosaccades	Antisaccades	Prosaccades	Antisaccades
Inhibitory fail probability	0.110; 0.150	0.057; 0.063	0.082; 0.106	0.086; 0.096
Inhibitory fail reaction time	1.580; 1.747	1.320; 1.437	1.523; 1.698	1.422; 1.521
Late prosaccade probability	0.963; 0.966	0.040; 0.046	0.935; 0.938	0.091; 0.100
Late prosaccade reaction time	2.870; 2.957	2.884; 2.928	3.169; 3.208	3.199; 3.257

## Discussion

In this article, we present a comprehensive framework for testing the utility of the antisaccade task in healthy young and older participants. We investigated age effects and test–retest reliability of directly measurable variables for prosaccade and antisaccade conditions: reaction time, error rate, saccade gain, and peak saccade velocity. We further decomposed the task with computational models and extracted computational model parameters, including inhibitory fail reaction time, inhibitory fail probability, late saccade reaction time, and late saccade probability.

As we had predicted, we found longer saccadic reaction times and significantly higher average error rates for older adults than for younger adults in the antisaccade task for both prosaccade and antisaccade conditions. Test–retest analysis for directly measurable variables revealed fair to excellent reliability, which indicated that these results are both representative and stable over time.

Furthermore, brain regions involved in controlling saccades are well characterized, and the underlying processes can be described by computational models (Heinzle et al., 2016). Hitherto, several computational models have been proposed that incorporate physiological mechanisms employing both an inhibitory mechanism and competition between action (Cutsuridis et al., 2007; Lo and Wang, 2016). A notable attempt was made to model the antisaccade paradigm by Noorani and Carpenter (2016). Their model consisted of three units racing to the threshold: an ANTI unit, a PRO unit, and a STOP unit. Noorani and Carpenter’s proposal is extended in two state-of-the-art computation models for the antisaccade task: the PROSA and SERIA models (Aponte et al., 2017). To the best of our knowledge, our study is the first to apply these computational models to investigating age differences and probe their test–retest reliability. These computational models extend the current understanding of processes that contribute to changes in reaction times and error rate and suggest that the changes can best be explained by faster or slower inhibition (Aponte et al., 2019). We used the PROSA and SERIA models (Aponte et al., 2017) to estimate latent processes that were not directly observable. Regardless of the age group, the SERIA model outperformed the PROSA model. Furthermore, our analysis of the SERIA model parameters revealed significantly more inhibition failures for older adults than for young adults. Additionally, older adults have significantly longer inhibitory fail reaction time, longer late saccade reaction time, and a higher probability of late saccades.

In addition to the preregistered hypotheses, we examined the reliability of the computational model parameters, which in the antisaccade condition exhibited fair to excellent ICC thresholds in both age groups.

### Test–retest reliability

One of the central goals of this study was to examine the test–retest reliability of all directly measurable behavioral variables. Adequate test–retest reliability is a prerequisite for compiling meaningful and suitable estimates for future longitudinal studies and identifying promising biomarkers for cognitive decline. For the older group of participants, all behavioral measures for the antisaccade and prosaccade conditions showed good to excellent reliability (0.59 < ICC < 0.89), so they are potential biomarkers for evaluating the healthy aging process. The behavioral measures for the younger group of participants for the antisaccade condition achieved 0.58 < ICC < 0.77, thus provided highly reliable results, especially for reaction time and error rate, whose reliability was excellent. However, for the prosaccade condition, in the younger group, we obtained slightly worse ICC scores. Notably, the reliability of the reaction time was still excellent. The lower reliability (ICC = 0.52) in the younger group’s prosaccades error rate is most probably explained by the fact that younger participants only performed errors in 1.3% of the trials. A possible explanation for this outcome is that the internationally standardized antisaccade protocol, which also addresses prosaccades, was established to enable clinical comparisons between neurologic and psychiatric conditions (Antoniades et al., 2013) and thus can be undemanding for healthy young participants.

Overall, the behavioral measures, in particular reaction time and error rate, produce very reliable outcomes over two recording sessions. However, saccade gain and peak saccadic velocity appear to be less reliable, especially for the prosaccade condition. Therefore, care should be taken when selecting the behavioral variables to be used for longitudinal studies or for tracking clinical progression in older patients. In summary, our study is in line with previous research that reported significant ICCs of measures for reaction times in prosaccade and antisaccade tasks and the antisaccadic direction errors (Klein and Berg, 2001; Ettinger et al., 2003; Klein and Fischer, 2005; Blekher et al., 2009). However, the test–retest intervals and the ages of specific groups of participants varied substantially across these studies. The 19-month test–retest correlations obtained in Klein and Fischer’s (2005) study ranged between 0.43 and 0.66 and suggested moderate reliability between test and retest during childhood and adolescence. Another study (Klein and Berg, 2001) found high test–retest correlations for all saccadic reaction times (ICC > 0.76). Nevertheless, these findings may be somewhat limited by sample size, as the study included only 20 healthy young participants.

The highest reliability (0.55 < ICC < 0.93) reported to date for reaction times and error rates was a study by Blekher et al. (2009) that evaluated the test–retest reliability of saccadic measures in prediagnostic carriers of the HD gene expansion and healthy controls within a one-month interval. They argued that the excellent reliability of saccadic latency and percentage of errors suggest that these measures could serve as potential biomarkers for evaluating the efficacy of neuroprotective agents in slowing or delaying HD’s progression. However, their sample included only 21 participants; thus, caution must be applied, because the findings might not be statistically robust. The variability in the ICCs reported in these studies can be also caused by specific task parameters such as the predictability of the condition, varying block size, and experimental setup.

To the best of our knowledge, our study reported the highest reliability for the antisaccade condition for reaction time, error rates, saccade gain, and peak saccadic velocity. This study extends knowledge of the reliability of behavioral measures for saccadic eye movements. The ICCs for an older group of participants emerged as good to excellent for most of our behavioral measures. Another strength of our study is that all reliability estimates presented here are based on large samples.

In addition, we have investigated the ICC for the four computational model parameters of the computational SERIA model. The reliability of the model parameters was fair to excellent in the antisaccade condition in both age groups. For inhibitory fail probability in the antisaccade condition, we achieved ICC = 0.78 for the younger group and ICC = 0.81 for the older group; excellent reliability. Moreover, the late prosaccade probability ICC score resulted in good reliability for the younger group (0.70) and fair reliability for the older group (0.53). Late prosaccade reaction time achieved excellent reliability in the older group (0.86) and fair reliability in the younger group (0.52). However, almost all measures displayed poor results (0.04 < ICC < 0.4) for the prosaccade condition except the late prosaccade reaction time for the younger group which resulted in ICC = 0.52.

However, the SERIA model was not primarily developed with data collected according to the standard protocol established by Antoniades et al. (2013) but with data from an another antisaccade paradigm studied in healthy young participants. The paradigm that was used to develop the SERIA model included three blocks of 192 randomly alternating prosaccade and antisaccade trials. The percentages of prosaccade trials in the three blocks were 20%, 50%, and 80%; thus, the participants could not predict whether each subsequent trial was an antisaccade or prosaccade trial. In contrast to the original study on which the SERIA model was developed, our participants did not exhibit enough errors in the prosaccades to obtain a stable estimate for the inhibition failures within a prosaccades condition. The reliance of SERIA on the internationally standardized antisaccades protocol means that this model should only be used and interpreted on the antisaccade condition. Therefore, further studies need to be undertaken on the computational models that take this straightforward paradigm into account.

### Age effects

The presence of age differences in reaction times, error rates, peak saccadic velocities, and saccade gains was investigated with a multivariate Bayesian generalized linear mixed model.

In agreement with previous research, the older group displayed higher error rates (Sweeney et al., 2001; Butler and Zacks, 2006) and reaction times (Crawford, 2017) in both conditions than did the younger group. Higher error rates and the consequently lower ability of older adults to voluntarily inhibit saccadic responses has been interpreted as an indicator of age-related inhibitory control decline (Raemaekers et al., 2006; Peltsch et al., 2011; Crawford et al., 2017). Moreover, the significant interaction for the error rate between the type of saccade and the age of the participant confirmed that aging effects are more substantial in the antisaccade condition and are connected to cognitive aging (Moschner and Baloh, 1994).

As suggested by the standardized protocol recommendations, we also compared metrics for saccadic eye-movement dynamics: saccade gain, that demonstrates the accuracy of eye movements relative to the displacement of stimuli and peak saccadic velocity. Our results are consistent with previous studies reporting no age-related differences in peak saccadic velocity (Zackon and Sharpe, 1987; Moschner and Baloh, 1994; Bono et al., 1996). Although a slight reduction in peak velocity was observed in the older age group, we did not establish any statistical significance for this result. These results indicate that the difference in reaction time is not attributed to the dynamics of saccadic eye movements but to underlying slower cognitive processing (Munoz et al., 1998). The saccade gain was lower in older participants than in younger ones, which is in agreement with Moschner and Baloh’s (1994) findings.

In addition to measures obtained from the multivariate model, the formal probabilistic computational model allowed us to analyze the age effects on four additional parameters.

The present study expands previous findings by showing that the SERIA model displays a considerably better model fit than the PROSA model in both younger and older participants. Thus, we conclude that changes in measurable reaction time and error rate can be explained by fast or slow inhibition and the probability of generating late voluntary prosaccades. This is different from the PROSA model, which cannot account for slow, voluntary prosaccades that have been observed in the antisaccade task (Lo and Wang, 2016).

Our results also revealed more inhibition failures, fast, reflexive prosaccades on prosaccade trials and errors on antisaccade trials, and late saccades. Late responses can trigger prosaccade and antisaccades with a certain probability (Aponte et al., 2017, 2019), higher for older adults than for younger adults. This is a further indicator of a reduction in inhibitory control in older adults (Sweeney et al., 2001). Moreover, older adults have significantly longer inhibitory fail reaction times and longer late saccade reaction times than younger people.

The biological interpretation of saccade inhibition in the antisaccade task has received much attention and is still debated (Schall et al., 2017). According to current theories, the inability to inhibit saccadic eye movements may be associated with age-related neurophysiological changes in the brain and with compensatory activation in frontal brain areas (Raemaekers et al., 2006; Peltsch et al., 2011; Crawford et al., 2017), including the visual cortex and the basal ganglia (DeSouza et al., 2003). Moreover, the impaired inhibitory control over saccades in older adults has been attributed to impaired function of the frontal lobes, but this notion is mainly based on findings from patients with lesions of the dorsolateral prefrontal cortex (Raemaekers et al., 2006; Peltsch et al., 2011; Crawford et al., 2017).

Neurophysiological recording studies have shown that a crucial step in the antisaccade task is the inhibition of saccade neurons in the frontal eye fields (Everling et al., 1997). This evidence has come from functional imaging and EEG studies. Further research should be undertaken to investigate the precise neural mechanisms required to inhibit the prepotent saccade.

In conclusion, we have described test–retest reliability and age-related differences in the performances of healthy younger and older participants in antisaccade tasks. The antisaccade task is relatively easy to measure and quantify and offers a window onto the very highest levels of cognitive functioning. Nevertheless, the current literature presents considerable variability in results and a lack of permament consensus regarding changes in antisaccade task performance in the lifespan. One way of addressing this problem was proposed by Antoniades et al. (2013): use a standardized protocol to enable comparison across different studies. Overall, the idea of a standardized protocol is appealing, and one that enabled comparisons between laboratories and clinics would be of great benefit. However, the protocol that was primarily established is for populations in advanced stages of neurodegenerative diseases or with considerable cognitive impairments. Our study has shown that the standardized protocol is more suitable for the older population than for healthy young participants, as indicated by excellent test–retest reliability in the older group. Moreover, the computational modeling revealed that only the model parameters from the antisaccade condition should be interpreted when using the standardized protocol. In future work, we aim to test the internationally standardized antisaccade protocol on the clinical group of patients diagnosed with mild cognitive impairment.

## Synthesis

Reviewing Editor: Satu Palva, University of Helsinki Decisions are customarily a result of the Reviewing Editor and the peer reviewers coming together and discussing their recommendations until a consensus is reached. When revisions are invited, a fact-based synthesis statement explaining their decision and outlining what is needed to prepare a revision will be listed below. The following reviewer(s) agreed to reveal their identity: Chrystalina Antoniades, Jaana Simola.

This paper presents a comprehensive framework to investigate the utility of the antisaccade task in testing inhibitory control processes among healthy young and older participants and to study the underlying processes of age-related changes in cognitive functions. Previous studies have largely overlooked the different sources of a worse performance among older as compared to younger participants. Age effects were investigated both by comparing the direct measures from the antisaccade task, e.g., reaction time, error rate, saccade gain and peak saccade velocity, as well as by parameters from two computational models. In addition, the authors examined the test-retest reliability of these measures. Test-retest reliability is an important prerequisite for using the antisaccade test in indentifying biomarkers for cognitive decline. A strong aspect of the current study is that all reliability estimates are based on large samples of participants.

It would be helpful for the readers to grasp the reliability of the results if Figures 3 and 7 would show the fits for each category of responses.

Figure 6. Shouldn't the title of panels C and D be the proportion of correct trials instead of Error rates?

In the abstract on line 7 please change young and old to younger and older, respectively, to be in line with your introduction.
